# Characterizing stroke presenting to a regional referral hospital before and during the COVID-19 pandemic in Uganda: a retrospective analysis

**DOI:** 10.1186/s12245-025-00830-6

**Published:** 2025-04-07

**Authors:** Josephine Nambi Najjuma, Timothy Mwanje Kintu, Jane Nakibuuka, Mark Kaddumukasa, Scovia N. Mbalinda, Martin Kaddumukasa, Christopher Burant, Shirley Moore, Martha Sajatovic, Edwin Nuwagira

**Affiliations:** 1 Faculty of Medicine, Mbarara University of Science and Technology, Mbarara, Uganda; 2 Department of Internal Medicine, College of Health Sciences, Makerere University, Kampala, Uganda; 3 Neurological and Behavioral Outcomes Center, University Hospitals Cleveland Medical Center, Case Western Reserve University School of Medicine, Cleveland, OH USA; 4 Department of Nursing, College of Health Sciences, Makerere University, Kampala, Uganda; 5 Frances Payne Bolton School of Nursing, Case Western Reserve University, Cleveland, OH USA

**Keywords:** Stroke, Sub-saharan Africa, COVID-19, Pandemic, Uganda

## Abstract

**Introduction:**

Stroke, a leading cause of global morbidity and mortality, disproportionately impacts low and middle-income countries, particularly in sub-Saharan Africa (SSA) which reports the highest stroke burden. The COVID-19 pandemic further complicated this situation, emerging as a significant stroke risk factor. The pandemic also disrupted healthcare systems worldwide, affecting stroke management and care accessibility, and leading to deteriorated conditions in stroke patients upon hospital admission. In this pre/during COVID-19 pandemic analysis of acute stroke cases presenting to a Ugandan hospital, we investigated the relationship between stroke admissions, management and treatment outcomes.

**Methods:**

This was a retrospective medical record review in which we analyzed medical charts of stroke patients admitted to Mbarara Regional Referral Hospital in 2019 (pre-COVID-19) and 2020 (during COVID-19). Socio-demographic data, stroke subtypes, medical history, and physical examination findings were extracted from the hospital records. Data analysis was performed using R-Studio, employing descriptive statistics and inferential analyses to compare stroke characteristics and outcomes across the two periods.

**Results:**

Data from 175 stroke patients was analyzed, with higher admission numbers in 2020 (69.7%), but a slightly higher mortality rate in 2019 as compared to 2020 (22.6% versus 18.9%, *p* = 0.711). A significant increase in acute ischemic stroke cases was observed in 2020, with no significant differences in stroke severity or functional ability between the two years. Clinical parameters such as admission oxygen saturation, blood sugar, temperature, and Glasgow Coma Scale (GCS) score, along with complications like aspiration pneumonia and infections, correlated with mortality. There was no significant difference in survival probability between pre- and during-pandemic periods. Admission GCS, pulse rate, and aspiration pneumonia were significant predictors of 14-day in-hospital mortality.

**Conclusions:**

The surge in acute ischemic stroke cases during the pandemic highlights the need for robust stroke care systems, especially in high-burden regions like SSA. Some key predictors of mortality are potentially modifiable, suggesting that early intervention and vigilant monitoring of risk parameters could improve survival rates. Findings also highlight the need for tailored care strategies and health system improvements especially during public health emergencies to enhance patient outcomes.

**Trial registration:**

Not Applicable.

## Introduction

Globally, stroke is among the leading causes of morbidity and mortality [1]. According to the world stroke association, the risk of stroke is higher in low and middle income countries (LMIC), with almost up to a three-fold greater rate of stroke incidence than western Europe and the USA [2]. Current trends indicate that sub Saharan Africa (SSA) has the highest stroke burden worldwide, with an incidence rate of up to 316 per 100 000, prevalence rates of 14 per 1000 population and 30 day mortality rate of up to 40% [1, 3].

In Africa, 25% of strokes occur in young people, 40% in middle-aged individuals, and 35% in the elderly. This high burden combined with grossly inadequate resources and limited access to interventions, has impacted the productivity of the young populations, where stroke risk factors extend beyond traditional ones such as hypertension, diabetes mellitus, and hyperlipidemia [4]. In contrast, high-income countries (HIC) are experiencing a reduction in both stroke burden and stroke-related mortality, thanks to advances in comprehensive stroke management [5].

The novel Corona virus disease 2019 (COVID-19) was identified as one of the risk factors for stroke especially among patients with severe forms of the disease [6]. Up to 3% of patients with severe forms of COVID-19 were reported to be at risk of stroke [7], as supported by some case reports from Africa [8, 9]. It was also reported that COVID-19 was more severe among patients with underlying medical conditions such as diabetes mellitus, hypertension and advanced age [10], all of which are also potential risk factors for stroke. The disruption in health care and global economic collapse affected the availability of resources for stroke management [11] as well as access to stroke care [12]. Additionally, there was reluctance of patients to present to hospital leading to delays in receiving appropriate care, especially in countries that had stringent lockdown measures such as Uganda [13]. Consequently, patients with stroke presented to hospital in a more deteriorated condition than expected. At Mbarara Regional Referral Hospital, it was anecdotally observed that the number of stroke patients admissions increased in 2020.

The lockdown in Uganda created major shifts in Uganda’s healthcare systems affecting patients’ access to health care [14], potentially including access to stroke preventive and management therapy. In this study of acute stroke cases presenting to a Ugandan hospital, we compared stroke admissions, management and treatment outcomes before and during lockdowns imposed during the COVID-19 pandemic. The information obtained will contribute in closing the knowledge gap in this relationship between COVID-19 and stroke, as well as informing stroke management during future public health crises.

## Methods

### Study design

This was a retrospective health record study that included two cohorts of stroke patients, those admitted in 2019 (before the COVID-19 first wave in Uganda) and those admitted in 2020 (during the COVID-19 pandemic). Demographic and clinical variables between pre and during pandemic cohorts were compared.

### Study setting

The study was carried out at Mbarara Regional Referral Hospital (MRRH), a teaching hospital for Mbarara University of Science and Technology and a tertiary institution that serves as a regional treatment center for Mbarara city and surrounding districts in Western Uganda. Adults with suspected stroke were screened at the Accident and Emergency unit where those confirmed to have a stroke by CT Scan results were admitted by resident doctors. Upon admission, all patient information was recorded in paper files. Upon discharge, the patient’s files were stored in the hospital registry/ records department.

### Study population

The study population consisted of medical records of patients who presented to MRRH during the study periods with a diagnosis of stroke as the final diagnosis at discharge. We included only medical records of patients above 18 years of age, who were not living with sickle cell disease and were not pregnant. Patients with sickle cell disease were excluded due to their distinct stroke risk profiles and unique management protocols, which could confound the analysis of stroke outcomes in the general population. Similarly, pregnant women were excluded because pregnancy introduces specific physiological changes and risk factors that may influence stroke risk and treatment outcomes differently from the non-pregnant population.

### Variables

We extracted socio-demographic characteristics, and stroke sub-types. Information on the medical history, including risk factors for stroke (such as history of diabetes, hypertension, atrial fibrillation, congestive heart failure, stroke, transient ischemic attack, myocardial infarction, dyslipidemia, seizures and current use of antihypertensive therapy, anticoagulant therapy, antiplatelet therapy, glucose-lowering therapy and lipid lowering therapy).

Physical examination findings include assessments of the level of consciousness using the Glasgow Coma Scale (GCS), stroke severity through the National Institutes of Health Stroke Scale (NIHSS), and functional ability and outcomes via the modified Rankin scale (mRS). Additionally, the Full Outline of UnResponsiveness (FOUR) score was utilized to evaluate the consciousness level in patients with severe neurological impairment, particularly beneficial for intubated or critical care patients [15]. The mRS, a widely accepted measure in stroke clinical trials, gauges the degree of disability or dependence in daily activities, ranging from 0 (no symptoms) to 6 (death), thus offering a comprehensive overview of a patient’s functional outcomes post-stroke [16].

Laboratory investigations findings recorded included: lipid profile, complete blood count (CBC), Blood sugar, electrocardiogram (EKG), echocardiograph (ECHO) and imaging investigations such as the CT scan results. The hospital disposition outcomes included: discharged, death, referral/transfer and absconding from treatment or unknown.

### Sample description

The study included all patients admitted at Mbarara Regional Referral Hospital in 2 key years, that is 2019 (before the COVID-19 pandemic in Uganda) and 2020 (during the COVID-19 pandemic). Only records for patients above 18 years and had a stroke diagnosis confirmed by a Computerized Tomography (CT) scan were included in this study.

### Data sources and management

The data collection tool was checked for completeness. Extraction of the de-identified data was carried out by three graduate nurses in November and December, 2022. The research assistants were trained by NNJ, the data collecting training was done using the recently discharged patient recorded that were not part of the study period. Each record was reviewed twice by two different research assistants and the findings compared for discrepancies and completeness. The double data entry process revealed no discrepancies. Consequently, no further discrepancy resolution methods or inter-rater reliability statistics were necessary. Back-up files of the database were kept at the end of each data entry session. All the data was kept on laptop with a password known to the study team as well as storage in cupboards under lock and key.

### Statistical analysis

Data was extracted into R-Studio version 4.1.0 and the same software was used for analysis. Descriptive statistics were used to report the rates of stroke admissions in the pre-COVID-19 pandemic year and during the COVID pandemic year (2020), summarize the participants’ socio-demographic characteristics, clinical and radiological classification of stroke and presented as tables. Categorical variables (such as gender, history of smoking) were expressed as proportions whereas continuous variables (such as age and admission GCS) were expressed as means with a standard deviation if normally distributed and, median with an inter-quartile range if skewed. The number of stroke admissions, subtypes of stroke and key clinical presentations (severity and functional ability) before and after the start of the COVID-19 lockdown (31st March, 2020) were then summarized.

To identify factors associated with in-hospital outcomes, continuous data was compared across groups using the T-test, and categorical data was compared using the Chi-square test. Survival analysis was conducted using the Kaplan-Meier method to estimate overall survival probabilities and compare survival distributions between groups. Overall survival probabilities were summarized at predefined time points (0, 3, 7, and 14 days). To assess differences in survival distributions between pre-pandemic and pandemic periods, Kaplan-Meier curves were stratified by year, and a log-rank test was performed to evaluate statistical significance.

Survival within the first 14 days was analyzed by censoring all observations with a time to event beyond 14 days. Kaplan-Meier curves and survival probabilities were summarized for this restricted time frame to focus on short-term outcomes. Sensitivity analysis was performed to assess the impact of missing data on survival estimates. Two approaches were used: (1) assuming missing observations were censored at the maximum observed follow-up time, and (2) imputing missing ‘time to event’ values with the median follow-up time. Findings were generated for both scenarios and compared with the original analysis to evaluate the robustness of the findings.

Cox proportional hazards regression was used to identify independent predictors of mortality. We set up the analysis so that for each variable, the reference category was that which we hypothesized to have the lowest risk of a mortality, for example, a history of stroke was hypothesized to be a predictor of mortality and was thus the reference range for that category was, “no history of stroke”. Prior to including the factors in the multivariable model, the variance inflation factor test was done to check for multi-collinearity. Because we used secondary data, we had variables with missing data. We established the proportion of missing data for each variable. Variables that had more than 30% of missing data were not included in the multivariate model.

The initial model included a broad set of explanatory variables, such as demographic factors (age, sex), stroke characteristics (subtype, stroke or TIA), clinical features (admission GCS, history of hypertension, other comorbidities), and complications (aspiration pneumonia, infections). A refined multivariable model focused on key predictors identified from the initial model, including stroke subtype, admission GCS, pulse rate, and infections (e.g., UTI). Hazard ratios (HRs) with 95% confidence intervals were calculated. All statistical inferential frameworks were based on the two-sided *p*-value and a 5% error margin.

## Results

### Participant characteristics

Data for 175 patients was extracted from the patient records, with 122 patients (69.7%) admitted in 2020. Majority were female (63%) and the overall in-hospital mortality rate was higher in 2019 (22.6%, 12/53) than in 2020 (18.9%, 23/122) (Table 1).

**Table 1:** Characteristics of the study participants presenting before and during the COVID-19 pandemic

Characteristic	Total (*n* = 175)	2019 (*n* = 53)	2020 (*n* = 122)
Age, mean (SD)	66.4 (14.8)	64.4 (14.7)	67.3 (14.9)
Gender (female)	110 (62.9)	29 (54.7)	81 (66.4)
Time to outcome (days)^1^, mean (SD)	48.9 (190.8)	87.1 (283.2)	34.1 (138.7)
Acute Ischemic stroke	84 (48.0)	21 (39.6)	63 (51.6)
Intracerebral hemorrhage	43 (24.6)	14 (26.4)	29 (23.8)
In-hospital mortality	35 (20.0)	12 (22.6)	23 (18.9)
History of alcohol use	71 (40.6)	25 (47.2)	46 (37.7)
History of smoking	47 (26.9)	15 (28.3)	32 (26.2)
History of hypertension	104 (59.4)	32 (60.4)	72 (59.0)
History of stroke or TIA	28 (16.1)	7 (13.2)	21 (17.4)

^1^ Time to outcome (days) refers to the duration, in days, from admission to hospital to the recorded outcome

### Stroke subtypes and severity, and characteristics of study participants before and during the COVID-19 pandemic

**Fig. 1:**
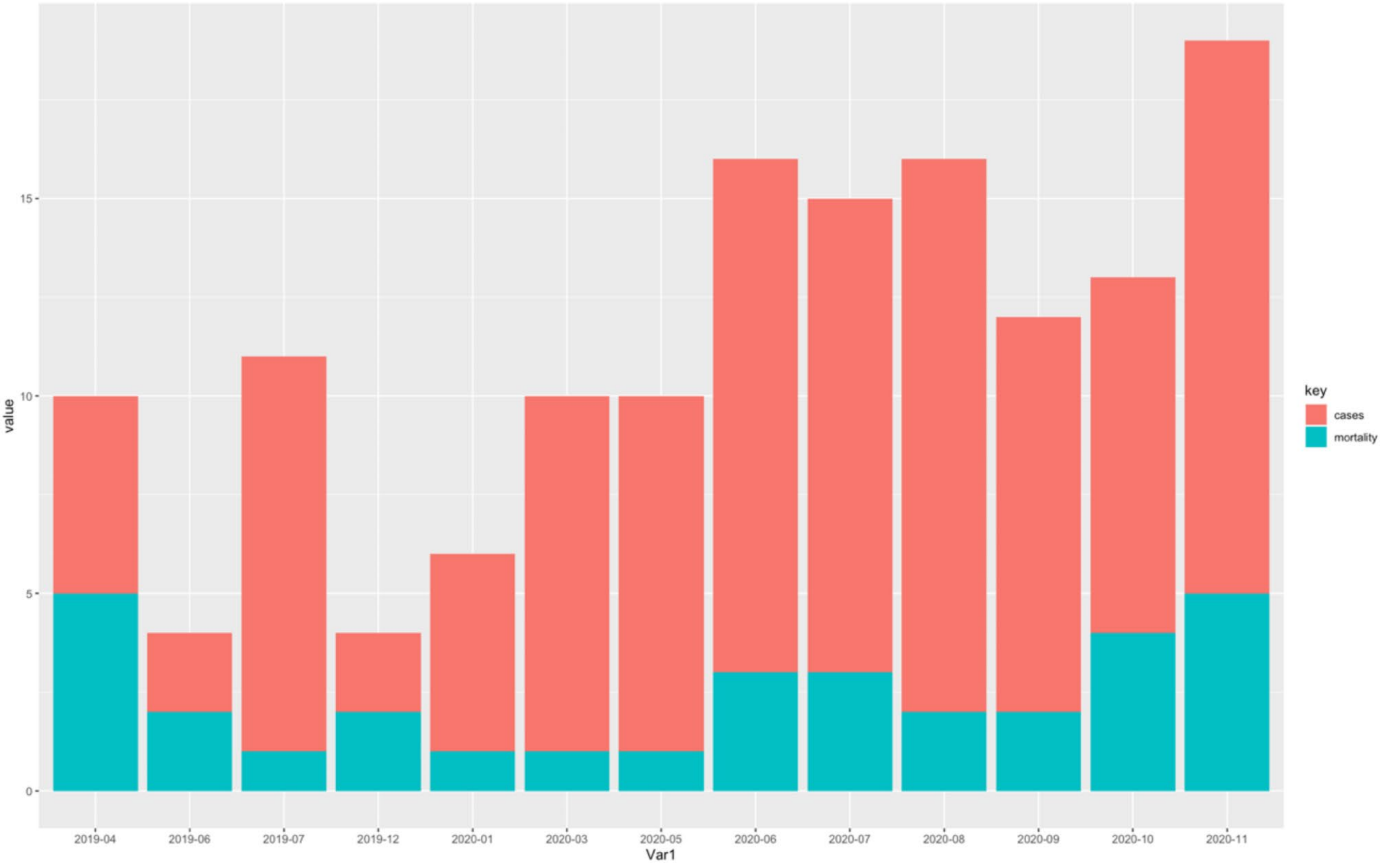
Changes in hospital admission and mortality before and during the COVID-19 pandemic. Stacked bar chart showing the monthly number of hospital admissions (red) and in-hospital deaths (teal) from April 2019 through November 2020. The horizontal axis (Var1) indicates the month and year, while the vertical axis (“value”) represents the count of admissions or deaths. Bars are subdivided by color to illustrate changes in admissions and mortality before and during the COVID‐19 pandemic. No specific references were consulted for this type of visualization

**Fig. 2:**
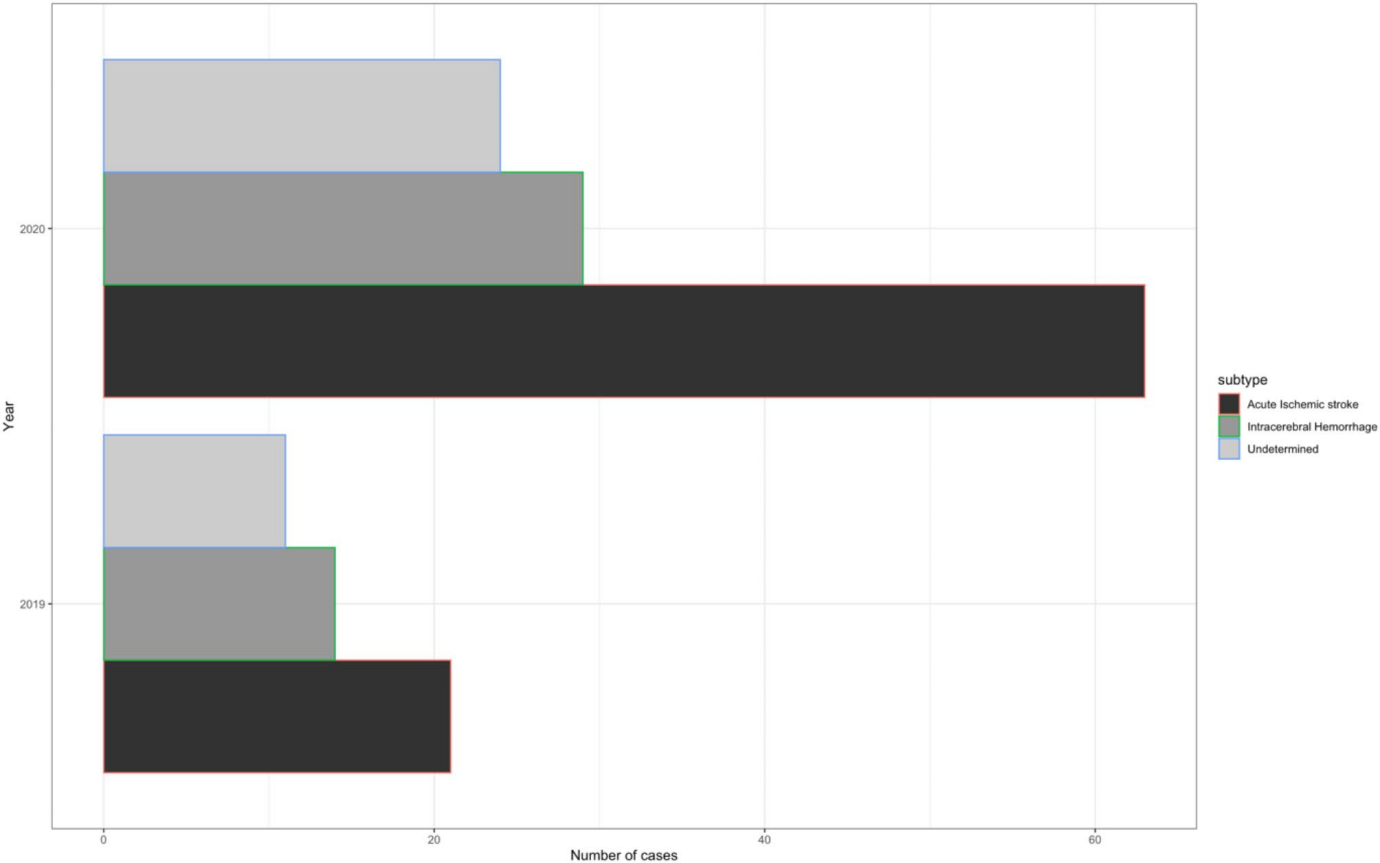
Stroke subtypes by year of admission. Bar chart illustrating the distribution of stroke subtypes by year of hospital admission. The horizontal axis represents the number of stroke cases, and the vertical axis indicates the admission year. Each color corresponds to a different stroke subtype: green for acute ischemic stroke, red for intracerebral hemorrhage, and blue for undetermined

**Fig. 3:**
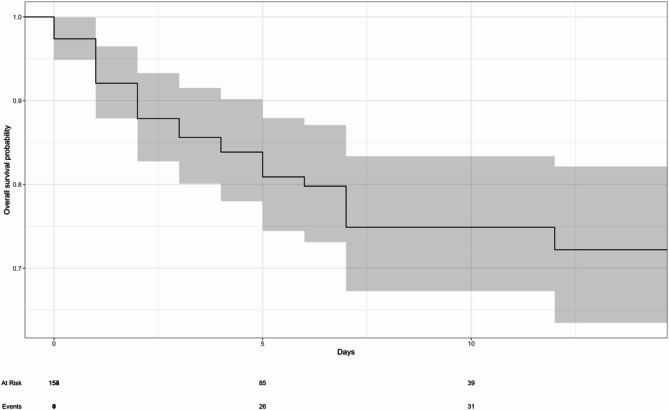
Kaplan-Meier Survival estimate showing overall 14-day mortality before and during the COVID-19 pandemic. Kaplan–Meier curve displaying the overall 14-day survival probability for all hospitalized patients before and during the COVID-19 pandemic. The solid black line shows the estimated survival, and the shaded region represents the 95% confidence interval. The x‐axis indicates days since hospital admission, and the y‐axis indicates the probability of survival. The table below the x‐axis shows the number of patients at risk (“At Risk”) and the cumulative number of deaths (“Events”) at each time point. Outcome data for 22 patients were not available due to incomplete records at the time of final data collection

There was a spike in the number of patients presenting to MRRH after May, 2020 (Fig. 1). The number of cases of acute ischemic stroke were observed to increase three-fold in 2020 as compared to 2019 (Fig. 2) and no significant differences in stroke severity or functional ability between 2019 and 2020 (Fig. 3).

### Comparison of outcomes based on clinical characteristics

Stroke subtype (*p* = 0.027), history of stroke (*p* = 0.005), history of a transient ischemic attack (*p* = 0.01), use of antiplatelet therapy (*p* = 0.005) or use of anticoagulant therapy (*p* = 0.016) prior to admission were found to be associated with the clinical outcome (alive or dead) among these patients. Additionally, the patients’ oxygen saturation on admission (*p* = 0.026), random blood sugar on admission (*p* = 0.045), temperature on admission (*p* = 0.04) and admission GCS were found to be associated with the clinical outcome (*p* < 0.001). Among the complications, aspiration pneumonia (*p* < 0.001) and infections (*p* = 0.024) were associated with poor outcomes. Participant’s score on the NIH severity stroke (*p* < 0.001) and mRS (*p* = 0.001) scales were also found to be associated with mortality (Table 2 and Table 3).

**Table 2:** Comparison of outcomes based on baseline characteristics and comorbidities both pre and during COVID-19 pandemic

Characteristic	Alive (*n* = 140)	Dead (*n* = 35)	Total (*n* = 175)	*p*-value	
Age, mean (SD)	66.5 (14.9)	66.2 (14.8)	66.4 (14.8)	0.913	
Female gender	90 (64.3)	20 (57.1)	110 (62.9)	0.557	
Acute ischemic stroke	74 (52.9)	10 (28.6)	84 (48.0)	**0.027**	
Intracerebral hemorrhage	32 (22.9)	11 (31.4)	43 (24.6)	**0.027**	
Undetermined stroke type^1^	24 (17.1)	11 (31.4)	35 (20.0)	**0.027**	
Time to outcome, mean (SD)^2^	60.9 (212.9)	3.1 (2.8)	48.9 (190.8)	0.127	
Complete physical examination done	111 (79.3)	22 (62.9)	133 (76.0)	0.07	
Admitted in 2019	41 (29.3)	12 (34.3)	53 (30.3)	0.711	
**Comorbidities**					
History of stroke	13 (9.3)	10 (28.6)	23 (13.1)	**0.005**	
History of transient ischemic attack	10 (7.1)	8 (22.9)	18 (10.3)	**0.01**	
History of smoking	37 (26.4)	10 (28.6)	47 (26.9)	0.903	
History of alcohol use	54 (38.6)	17 (48.6)	71 (40.6)	0.342	
Diabetes	21 (15.0)	8 (22.9)	29 (16.6)	0.388	
Hypertension	80 (57.1)	23 (65.7)	103 (58.9)	0.466	
History of Atrial Fibrillation	4 (2.9)	4 (11.4)	8 (4.6)	0.088	
Prior myocardial infarction	7 (5.0)	4 (11.4)	11 (6.3)	0.329	
History of Dyslipidemia	133 (95.0)	30 (85.7)	163 (93.1)	0.426	
Living with HIV	11 (7.9)	2 (5.7)	13 (7.4)	1	
Current use of anticoagulant therapy	10 (7.1)	8 (22.9)	18 (10.3)	**0.016**	

^**1**^ 13 cases were missing and recorded as unknown neuroimaging results

^2^ Time to outcome (days) refers to the duration, in days, from admission to hospital to the recorded outcome

**Table 3:** Comparison of hospital outcomes based on admission vital signs, complications and functional outcomes

Characteristic	Alive (*n* = 140)	Dead (*n* = 35)	Total (*n* = 175)	*p*-value
**Admission Vital Signs**				
Pulse rate	84.3 (18.5)	92.3 (24.0)	86.0 (20.0)	**0.037**
Respiratory rate	24.3 (22.4)	24.6 (6.9)	24.3 (20.5)	0.948
Oxygen saturation	0.9 (0.1)	0.9 (0.1)	0.9 (0.1)	0.026
Temperature	36.6 (0.7)	75.3 (174.3)	44.3 (77.6)	**0.041**
GCS	12.4 (2.4)	8.1 (3.3)	11.5 (3.2)	**< 0.001**
Random blood sugar	8.8 (6.8)	13.7 (14.3)	9.7 (8.8)	**0.045**
**Complications**				
Dementia	3 (2.1)	3 (8.6)	6 (3.4)	0.183
Aspiration pneumonia	28 (20.0)	21 (60.0)	49 (28.0)	**< 0.001**
Hypoglycemia	3 (2.1)	3 (8.6)	6 (3.4)	0.28
Pressure sores	7 (5.0)	2 (5.7)	9 (5.1)	1
Electrolyte Imbalances	28 (20.0)	11 (31.4)	39 (22.3)	0.762
Hyperglycemia	35 (25.0)	12 (34.3)	47 (26.9)	0.723
Other infections	34 (24.3)	16 (45.7)	50 (28.6)	**0.024**
**Functional Outcomes**				
National Institute of Health Stroke Scale	10.9 (4.1)	16.1 (5.5)	11.3 (4.4)	**0.001**
FOUR Score	32.3 (165.1)	5.8 (5.4)	30.7 (160.2)	0.697
mRS	3.9 (0.9)	5.8 (0.6)	4.1 (1.1)	< 0.001

### In-hospital mortality and associated factors

#### Trends of survivor of 14-day in-hospital mortality among stroke patients

**Fig. 4:**
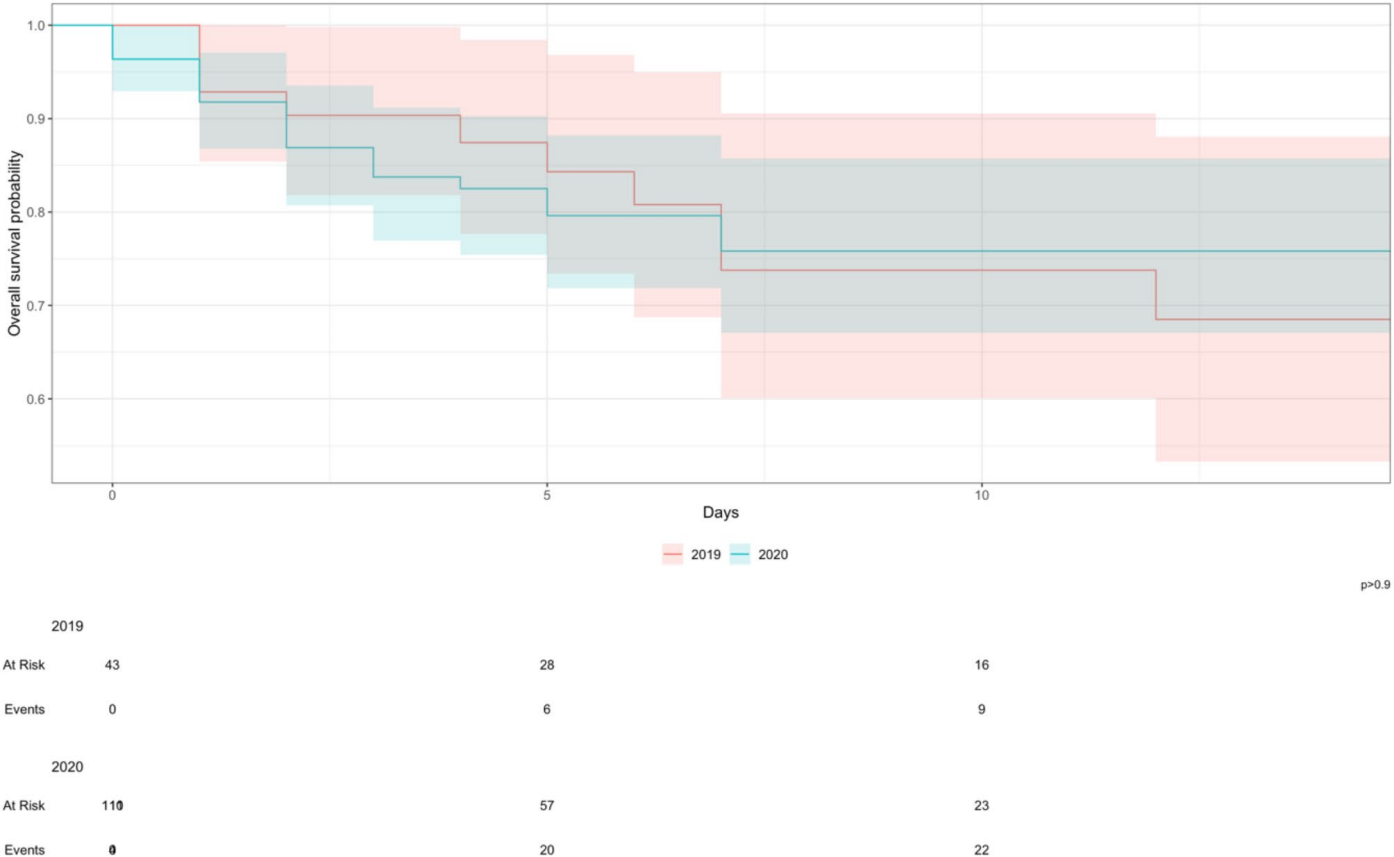
Kaplan-Meier Survival estimate comparing 14-day mortality before and during the COVID-19 pandemic. Kaplan–Meier curves illustrating 14-day survival probabilities for patients admitted in 2019 (red line) versus 2020 (teal line). The shaded regions around each line denote 95% confidence intervals for the survival estimates. The x‐axis shows days since admission, and the y‐axis indicates the estimated probability of survival. The tables below the x‐axis list the number of patients “at risk” (still alive and under observation) and the cumulative number of deaths (“events”) for each group at the indicated time points. The p‐value (> 0.9) reflects a log‐rank test indicating no statistically significant difference between the two survival curves

All patients who died were noted to die within the first 14 days of admission. The probability of patients’ survival after the 1st, 3rd, 7th, and 14th days after admission was 97.4%, 85.7%, 75.1%, and 72.5%, respectively. The probability of survival declined as follow-up time increased (Fig. 4). When stratified by period, the probability of patient survival was higher before the pandemic compared to during the pandemic on the 1st day (100% vs. 96.4%), 3rd day (90.3% vs. 83.9%). However, survival was higher during the pandemic compared to before the pandemic on the 7th day (76.1% vs. 73.8%) and the 14th day (76.1% vs. 68.5%). The differences in survival between the two periods were not statistically significant (*p* = 0.90) (Fig. 4).

##### Sensitivity analysis

To assess the robustness of the survival estimates, sensitivity analyses were conducted to account for 21 patients with missing data. Missing observations were assumed censored at the maximum follow-up time. The survival probabilities under this assumption were consistent with the main analysis: 97.7%, 87.5%, 79.0%, and 77.4%, respectively at 1, 3, 7, and 14 days, respectively. Comparing survival estimates between the main analysis and sensitivity analyses demonstrated that the missing data had minimal impact on the overall survival probabilities. Additionally, the predictors of mortality (admission GCS, pulse rate, and aspiration pneumonia) remained significant across sensitivity analyses, suggesting the robustness of the findings.

### Predictors of in-hospital mortality among stroke patients

Multivariable Cox regression analysis identified significant predictors of 14-day in-hospital mortality. Patients with higher admission GCS had lower mortality (aHR: 0.65, CI: 0.47–0.89, *p* = 0.007), while higher admission pulse rate (aHR: 1.04, CI: 1.01–1.07, *p* = 0.023) and aspiration pneumonia (aHR: 5.10, CI:1.07–24.18, *p* = 0.040) were associated with increased mortality (Table 4).

**Table 4:** Predictors of in-hospital mortality among stroke patients during the COVID-19 pandemic at bivariate and multivariate analysis

Characteristic	Description	Hazard Ratios (HR)	adjusted Hazard Ratios (aHR)
Age	Mean (SD)	0.99 (0.97–1.02, *p* = 0.636)	-
Gender	Female	0.66 (0.28–1.54, *p* = 0.333)	-
Stroke subtype	Acute ischemic stroke	-	-
	Intracerebral hemorrhage	1.76 (0.59–5.26, *p* = 0.308)	3.21 (0.72–14.30, *p* = 0.126)
	Undetermined	**4.17 (1.55–11.22**,***p*** **= 0.005)**	0.88 (0.18–4.31, *p* = 0.873)
Complete physical examination	Not done	2.16 (0.90–5.16, *p* = 0.083)	-
Alcohol use	Yes	2.34 (0.97–5.67, *p* = 0.058)	-
Smoking	Yes	1.54 (0.61–3.92, *p* = 0.363)	-
History of hypertension	Yes	1.09 (0.45–2.63, *p* = 0.847)	-
History of stroke or tia	Yes	**2.69 (1.09–6.67**,***p*** **= 0.033)*p*** **= 0.033)*p*** **= 0.033)**	0.93 (0.20–4.30, *p* = 0.930)
Admission Pulse rate	Mean (SD)	**1.02 (1.00-1.04**,***p* = 0.048)**	**1.04 (1.01–1.07**,***p*** **= 0.023)**
Admission SPO2	Mean (SD)	0.03 (0.00-1.10, *p* = 0.056)	-
Admission GCS	Mean (SD)	**0.69 (0.60–0.79**,*p* **< 0.001)**	**0.65 (0.47–0.89**,***p* = 0.007)**
Aspiration pneumonia	Yes	**5.79 (2.30-14.53**,*p*** < 0.001)**	**5.10 (1.07–24.18**,***p* = 0.040)**
Other infections	Yes	**3.38 (1.40–8.15**,***p*** **= 0.007)**	0.47 (0.09–2.59, *p* = 0.388)

## Discussion

The aim of this study was to compare the rate of stroke admissions, subtypes of stroke and key clinical presentations (severity and functional ability) among stroke patients presenting to Mbarara Regional Referral Hospital in south-western Uganda in 2019 before the COVID-19 pandemic as compared to 2020 during the COVID-19 pandemic. Additionally, this study identified factors associated with 14-day in-hospital outcomes among patients with stroke during the COVID-19 pandemic.

Majority of the patients enrolled in this study were admitted in 2020 (69.7%) as compared to 2019, with a spike in cases seen after June, 2020. This is contradictory to some previous studies that reported a decrease in stroke volume following the COVID-19 pandemic [17, 18]. one study reported an increase in calls for stroke to the emergency department but fewer admissions for stroke [19]. Additionally, there was an increase in the proportion of cases of acute ischemic stroke from 39.6% in 2019 to 51.6% of the total stroke cases in 2020. This could be due to the reported association of COVID-19 increasing the risk of thromboembolic diseases, such as acute ischemic stroke [20], especially in younger individuals [21].

The overall 14-day survival rate of stroke patients in this study was 72.5%. The 14-day survival rate during the pandemic in 2020 (76.1%) was higher than in 2019 before the pandemic (68.5%). However, this difference in survival rate was not statistically significant. Additionally, there was no difference in the NIHSS score (a measure of stroke severity) before and during the COVID-19 pandemic in this setting, despite a previous report of an increase in stroke severity during the pandemic [22]. Potential factors influencing these findings could include changes in patient health seeking behavior during lockdowns, including delays in seeking care [23]. These factors may have impacted the stroke population presenting to the hospital.

The mRS is a global outcomes rating scale for patients’ post-stroke [24]. In this study, patients admitted in 2020 had a higher average mRS as compared to those admitted in 2019. This is in line with previous findings from a study in India that reported that COVID-related strokes were more severe and associated with higher morbidity and mortality [25]. However, none of the participants in our cohort had confirmed comorbid COVID-19. Despite no significant difference in the 14-day survival rate and stroke severity (NIHSS score) between the pre-pandemic and pandemic periods, a higher average mRS in 2020 suggests increased post-stroke morbidity during the pandemic. This observed difference between NIHSS and mRS scores can be explained by the distinct aspects these scales measure. While the NIHSS focuses on stroke severity at presentation, the mRS evaluates functional outcomes and post-stroke disability, often reflecting the long-term impact of the stroke. As aforementioned, during the pandemic, factors such as delayed healthcare access, disruptions in post-stroke rehabilitation, and the overall strain on health systems might have contributed to worse functional outcomes, as indicated by higher mRS scores, despite similar NIHSS scores. Additionally, selective admission of patients with higher baseline dependency or pandemic-related vulnerabilities could explain this disparity.

The overall in-hospital mortality in this study was 20% which is lower than that from a study done at MRRH in stroke patients admitted between 2014 and 2015 (38%) [26]. However, this in-hospital mortality was higher than that identified in a systematic review by Mohammed et al. [27] that reported 15% in-hospital mortality in Eastern Africa. This disparity in mortality rates could be explained by the differences in clinical characteristics of participants included in the systematic review.

The commonest complications in this cohort of stroke patients were aspiration pneumonia, hyperglycemia, urinary tract infections, and electrolyte imbalances. Impaired swallowing mechanism due to neurological injury has been noted in almost half of stroke patients previously been diagnosed with pneumonia [28]. In this study, 28% of stroke patients experienced aspiration pneumonia, with 34% of patients admitted in 2019 experiencing aspiration pneumonia compared to 25% of patients admitted in 2020. Additionally, aspiration pneumonia was identified as a significant predictor of 14-day in-hospital mortality in these patients, consistent with other hospital-based studies [29–31]. Current guidelines have suggested evaluation by a speech-language pathologist (SLP) for all patients suffering from stroke to enable timely recognition of high-risk patients [28]. However, SLPs are not readily available in low-resource settings. Training nurses or other medical staff to assess swallowing impairment is a potential opportunity to mitigate the risk of aspiration pneumonia in these patients.

Patients’ admission GCS and pulse rate were also identified as predictors of mortality. Admission GCS has previously been found to be a predictor of 30-day mortality in a previous study done in an urban part of Uganda [32]. Despite the postulated effects of the lockdown delaying time to presentation at the emergency department and thereby affecting the severity of stroke at admission, there was no significant difference in GCS score on admission pre and during the COVID-19 pandemic. The admission heart rate has previously been identified as a predictor of mortality in stroke [33], although the cited study focused on atrial fibrillation-related strokes. However, findings from the PRoFESS trial also indicated that heart rate was a risk indicator among patients with stroke and a lower heart rate was associated with a better functional outcome in patients with ischemic stroke [34]. This emphasizes the need to update clinical management protocols to provide close monitoring and control of heart rate in order to ensure optimal outcomes in stroke patients.

This study has a few limitations that should be acknowledged. First, the study was conducted based on records from a single site (a regional referral hospital in south-western Uganda), limiting the generalizability of the findings to other regions or healthcare settings. Additionally, we did not confirm COVID-19 status in the study participants, which prevents us from directly assessing the impact of COVID-19 infection on stroke incidence and outcomes. The retrospective nature of the study also introduced potential biases, including a reliance on medical records for data collection, which were incomplete or inconsistent. Finally, the absence of data on time from symptom onset to hospital presentation precludes an analysis of whether delays in care might have happened and how these delays in care might have impacted outcomes. Despite these limitations, the study provides valuable insights into stroke management during the COVID-19 pandemic and highlights areas for improvement in resource-limited settings. Future studies addressing these limitations could further advance our understanding of stroke care in resource-limited contexts.

## Conclusion

The study demonstrated a significant increase in stroke cases, in particular ischaemic strokes admitted to MRRH during the start of the COVID 19 pandemic globally and in Africa. It provides insights into stroke management during a global health crisis. The reported overall in-hospital mortality rate of 20% is higher than regional averages, highlighting the ongoing challenges in stroke care in this setting. The study identified significant risk factors on admission for mortality including impaired GCS, aspiration pneumonia and pulse rate, some of which are modifiable. It is therefore recommended that healthcare systems in similar settings prioritize training for healthcare professionals in stroke care, particularly in the early identification and management of swallowing impairment and aspiration pneumonia and monitoring of vital signs. Additionally, we suggest updating clinical management protocols to include close monitoring and control of heart rate for optimal stroke outcomes.

## Data Availability

The datasets used and/or analyzed during the current study are available from the corresponding author(s) upon reasonable request.
